# Aneurysmal subarachnoid hemorrhage: predictive score for angiographic vasospasm

**DOI:** 10.1055/s-0046-1829045

**Published:** 2026-10-02

**Authors:** Mariana Duarte Gomes, Henrique Manuel Campos Cardoso, João Saraiva, Miguel Quintas-Neves

**Affiliations:** 1 Unidade Local de Saúde de Braga, Departamento de Neurorradiologia, Braga, Portugal.; 2 Universidade do Minho, Escola de Medicina, Instituto de Investigação em Ciências da Vida e Saúde (ICVS), Braga, Portugal.; 3 Universidade do Porto, Faculdade de Medicina, Porto, Portugal.

**Keywords:** Subarachnoid Hemorrhage, Multidetector Computed Tomography, Cerebral Hemorrhage, Vasospasm, Intracranial

## Abstract

**Background:**

Aneurysmal subarachnoid hemorrhage is a condition resulting from the rupture of a cerebral aneurysm, sometimes leading to delayed cerebral ischemia, primarily due to angiographic vasospasm; its prediction is crucial to improve patient management and outcomes.

**Objective:**

To assess the predictive value of clinical, analytical and imaging parameters for vasospasm in aneurysmal subarachnoid hemorrhage and to develop a clinical practical score.

**Methods:**

The current single-center retrospective study included 115 patients with aneurysmal subarachnoid hemorrhage who underwent a computed tomography scan within 24 hours of ictus. Clinical and analytical data were collected, and Hounsfield unit (HU) measurements were independently assessed. Statistical analyses were conducted to identify predictors of vasospasm.

**Results:**

Vasospasm occurred in 40% of patients (46/115). The multivariate analysis identified intracerebral hemorrhage (odds ratio [OR] = 2.482; 95%CI = 1.019–6.046; *p* = 0.045), interpeduncular HU value (per 5 HU increase: OR = 1.253; 95%CI = 1.044–1.504; *p* = 0.016), and serum chloride ≥ 106 mEq/L (OR = 3.545; 95%CI = 1.457–8.621; *p* = 0.005) as independent predictors ( *p* < 0.05) of vasospasm. The model, combined as a simplified score including these 3 variables, demonstrated excellent discriminative capacity (area under the curve [AUC] = 0.886; bootstrapped AUC = 0.885).

**Conclusion:**

We identified significant predictors of vasospasm in patients with aneurysmal subarachnoid hemorrhage, namely interpeduncular HU value, intracerebral hemorrhage, and serum chloride levels. A practical 5-point score based on these factors offers a potentially-robust, easy-to-use tool for early risk stratification, which may guide personalized follow-up and timely interventions. Further prospective multicenter studies are recommended to validate these findings.

## INTRODUCTION

Aneurysmal subarachnoid hemorrhage (aSAH) is a severe neurological emergency resulting from the rupture of an intracranial aneurysm, leading to bleeding into the subarachnoid space. 1 2 While trauma is the most common cause of subarachnoid hemorrhage, aneurysmal rupture accounts for approximately 85% of non-traumatic cases. 3 Despite a global decline in the incidence of subarachnoid hemorrhage, the mortality rate for aSAH remains alarmingly high, with 30 to 40% of the patients dying within the first month, and many survivors experiencing significant neurological deficits. 3 4 5

Aneurysms responsible for aSAH are typically located at arterial bifurcations within the circle of Willis, where hemodynamic stress contributes to their formation. The rupture of these aneurysms initiates a cascade of events (including increased intracranial pressure, irritation of the meninges, and the release of blood breakdown products into the cerebrospinal fluid), which triggers inflammatory responses. These processes are central to developing secondary complications, such as cerebral vasospasm. 3 4 5 6

This vascular complication can be classified as either *angiographic* , characterized by arterial narrowing visible on imaging without necessarily correlating with symptoms, or *symptomatic* , in which clinical signs of neurological deterioration occur. The current study focuses on the angiographic form, which typically peaks between 3 and 14 days after ictus, and can significantly reduce cerebral blood flow, leading to delayed cerebral ischemia and potential permanent neurological deficits. 3 7 8

Given these concerns, identifying reliable vasospasm predictors is crucial to accurately select high-risk groups and ensure they are subjected to specific follow-up protocols. Among the most consistent imaging-based predictors in the literature are the extent of subarachnoid blood, as quantified by the Modified Fisher Scale, 3 and Hounsfield Units (HU) in specific locations such as the interpeduncular cistern, where blood accumulation is typically more pronounced. However, these predictors have yet to achieve consensus and are not fully established in the clinical practice. 9 10

Additionally, clinical factors such as the patient's condition upon admission, particularly poorer clinical grades, as assessed by scales such as the Hunt and Hess Scale or the World Federation of Neurological Surgeons Scale, have been suggested to be associated with an increased likelihood of vasospasm. Similarly, biochemical indicators, particularly inflammatory markers and ionic disturbances such as leukocytosis and hyponatremia, have been linked to a higher risk of vasospasm, though further validation is needed. 11

Despite these advancements, the multifactorial nature of vasospasm, involving complex inflammatory responses, blood breakdown products, and prolonged arterial contraction, complicates the use of a single predictor. Although extensive research has explored potential vasospasm markers, most are evaluated in isolation and have limited applicability in routine clinical practice. Consequently, there remains a lack of simple and reproducible tools that integrate readily-available clinical, imaging, and laboratory parameters for early risk stratification. Therefore, the present study aims to develop a robust predictive score incorporating these factors to guide targeted follow-up and timely interventions in high-risk patients, with the potential to improve outcomes and reduce morbidity and mortality.

## METHODS

### Study design and population

We conducted an observational, single-center, retrospective study of patients with subarachnoid hemorrhage admitted to a tertiary hospital between January 2010 and June 2020 ( *n* = 370). The patients were excluded if the subarachnoid hemorrhage was secondary to causes other than aneurysmal rupture, including traumatic subarachnoid hemorrhage, cerebral amyloid angiopathy, reversible cerebral vasoconstriction syndrome, carotid stenosis, perimesencephalic hemorrhage, cerebral venous thrombosis, hypertensive hemorrhage, intracranial neoplasm, or arteriovenous malformations. Additional exclusion criteria were applied based on imaging availability and clinical relevance to the study objectives. Specifically, cases were excluded if the admission non-contrast computed tomography (CT) scan was performed more than 24 hours after symptom onset, as delayed imaging could affect hemorrhage-density measurements, or if the admission CT was unavailable. Patients who died within the first 7 days after hemorrhage were excluded because angiographic vasospasm could not be reliably assessed, as were patients who underwent radical surgery more than 14 days after symptom onset. Cases with incorrect diagnostic coding were also excluded. Furthermore, patients without visible blood in the interpeduncular cistern were excluded, as HU measurement was not feasible. After applying these inclusion/exclusion criteria, the final sample consisted of 115 patients ( Figure 1 ). Missing data were handled using a complete-case approach; patients with missing imaging or laboratory data required for the analyses were excluded from the final study population.

**Figure 1:**
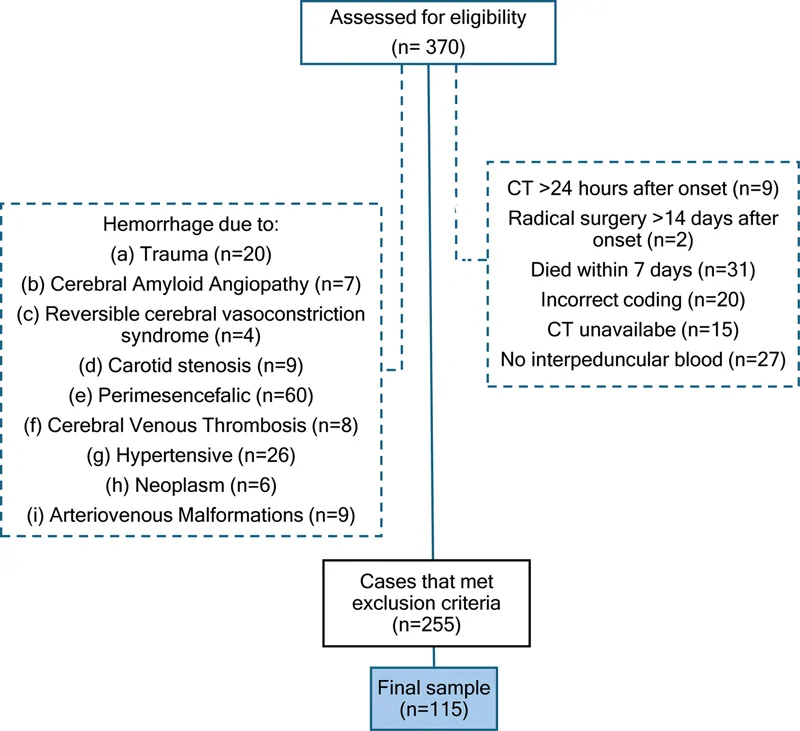
Patient enrollment flowchart.

### Ethical considerations

The current study was approved by the Ethics Committee for Health and the Data Protection Office of our hospital. Due to the retrospective nature of the study, formal written consent was deemed unnecessary by the Ethics Committee.

### Data collection

Clinical and imaging data were extracted from digital medical records and anonymized in a Microsoft Excel (Microsoft Corp.) worksheet created by the main investigator.

### Clinical data

The clinical data collected included the date of the admission CT scan, patient age, sex, aneurysm location, Hunt & Hess and modified Fisher grades, aneurysm treatment type, presence or absence of vasospasm (confirmed by an angiographic study), day of vasospasm onset, and whether it was asymptomatic or symptomatic. Data on laboratory blood tests (hemoglobin, neutrophils, leukocytes, platelets, C-reactive protein, and electrolyte panel: sodium, potassium, and chloride) were collected within 24 hours of admission. In accordance with the European Stroke Organization guidelines for the management of intracranial aneurysms and subarachnoid hemorrhage, 12 all patients received oral nimodipine 60 mg every 4 hours, starting within 96 hours of aSAH and continuing for 21 days. If vasospasm was detected, rescue therapies, such as intravenous milrinone or endovascular verapamil, were also administered. Electrolyte imbalances were assessed every 12 to 24 hours during the first 72 hours, along with continuous fluid status monitoring to prevent dehydration or overload complications. Acute hypertension on admission was invariably treated, to reduce the risk of rebleeding. 3

### Imaging evaluation

The HU were measured on the axial plane of the admission CT scan by drawing a circular region of interest (of 5 mm ^2^ ) in the interpeduncular cistern, to minimize partial volume effects and accurately evaluate the hematoma density, as illustrated in Figure 2 . Two independent raters with 8 and 5 years of neuroradiology experience respectively, blinded to clinical data, performed these measurements using the Picture Archiving and Communication System – SECTRA (Sectra AB) software. Most scans were performed on an Aquilion TSX-101A (16 slices; Toshiba Medical Systems Corporation), with 5 performed on other scanners for patients initially admitted elsewhere. Slice thicknesses were recorded due to their heterogeneity.

**Figure 2:**
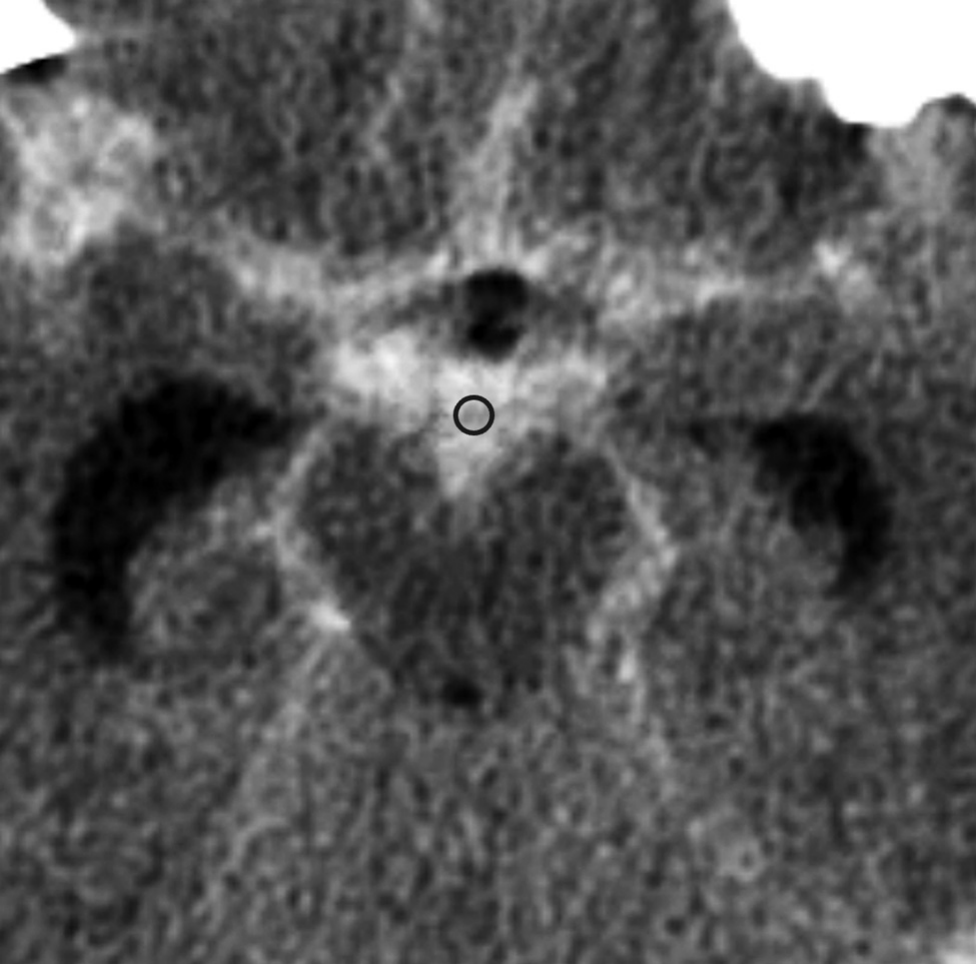
Axial section of a brain computed tomography (CT) scan at the level of the interpeduncular cistern, demonstrating diffuse aneurysmal subarachnoid hemorrhage, and the measurement of the Hounsfield units using a region of interest (ROI) of 5 mm ^2^ .

### Definition of outcomes

The primary outcome was the occurrence of angiographic vasospasm, defined as a segmental or diffuse narrowing of the main cerebral arteries of at least 30%, 13 14 compared with baseline (that is the admission CT angiography scan), as visualized on digital subtraction angiography or follow-up CT angiography, occurring between days 4 and 14 following aSHA. The secondary outcomes included the association between angiographic vasospasm and:

- imaging findings, specifically blood accumulation in the interpeduncular cistern (in HU) on the admission CT scan and presence of intracerebral hemorrhage (ICH), defined as a parenchymal hematoma visible on the admission non-contrast CT scan, distinct from subarachnoid or intraventricular hemorrhage;
- laboratory parameters, including serum electrolyte levels (sodium and chloride), leukocyte count, and C-reactive protein; and
- clinical factors, such as the score on the Hunt & Hess Scale upon admission.

### Statistical analysis

The statistical analysis was performed using IBM SPSS Statistics for Windows (IBM Corp.) software, version 29.0.0. The continuous variables were expressed as mean ± standard deviation or median and interquartile range (IQR) values, as appropriate. Differences in baseline clinical characteristics between patients with and without vasospasm were analyzed using unpaired *t* -tests or Mann-Whitney U tests. The categorical variables were expressed as frequencies. Interobserver agreement for interpeduncular HU measurements was assessed using intraclass correlation coefficients (ICCs), and the mean value of both measurements was used for analysis when agreement was significant. The association between angiographic vasospasm and each assessed variable was initially evaluated using univariate logistic regression. Variables showing a *p* -value ≤ 0.05 in the univariate analysis were subsequently entered into a multivariate logistic regression model to identify independent predictors of vasospasm.

For the continuous variables of interest, including interpeduncular HU values and serum chloride levels, optimal cutoff values were determined using receiver operating characteristic (ROC) curve analysis, selecting thresholds that maximized sensitivity and specificity. The interpeduncular HU cutoff (> 44 HU) was also consistent with values previously reported in the literature. 9

Score development was based on the relative magnitude of the Beta (β) coefficients derived from the multivariate logistic regression model, with higher coefficients receiving greater weight to preserve proportional risk contribution. The final predictive score ranged from 0 to 5 points. Because only 1 patient had a score of 0, this category was merged with score of 1 for subsequent analyses to improve model stability and ensure the reliable estimation of vasospasm risk across score categories. Multicollinearity among variables included in the multivariate logistic regression model was assessed by examining correlation matrices and variance inflation factors, and no evidence of significant collinearity was identified. Missing data were handled using a complete-case approach for each analysis. Patients with missing imaging or laboratory data required for a given analysis (such as serum chloride levels for score development) were excluded from that specific analysis.

The discriminative power was assessed using the ROC curve analysis and validated internally using non-parametric bootstrapping with one thousand iterations. Score performance was further explored by calculating the observed probability of vasospasm for each score value. Values of *p* < 0.05 were considered statistically significant. The ideal sample size was calculated using the G*Power (free) software, V3.1.9.7, resulting in a minimum requirement of 189 patients.

## RESULTS

The baseline characteristics of the 115 patients with aSAH are summarized in Table 1 . Vasospasm developed in 46 patients (40.0%), 17 of whom (14.8%) were under 50 years of age, 14 (12.2%), between 50 and 65, and 15 (13.0%) over 65 years. Female patients comprised 69,6% (80 patients) of the cohort.

**Table 1:** Baseline characteristics of patients with aneurysmal subarachnoid hemorrhage tested for vasospasm

**Variables**	**All patients**	**No vasospasm**	**Vasospasm**	***p*** **-value**
	**(** ***n*** ** = 115)**	**(** ***n*** ** = 69)**	**(** ***n*** ** = 46)**	
**Age (in years): n (%)**				
< 51	39 (33.9)	22 (19.1)	17 (14.8)	X ^2^ (2) = 0.484; *p* = 0.784
51–65	39 (33.9)	25 (21.7)	14 (12.2)	
> 65	37 (33.9)	22 (19.1)	15 (13.0)	
**Female sex: n (%)**	80 (69.6)	48 (41.7)	32 (27.8)	X ^2^ (1) = 0.004; *p* = 0.947
**Mean interpeduncular HU values**	48 ± 11	46 ± 2	51 ± 1	t (107.99) = 3.013; *p* = ** 0.002 ^a^**
**Mean slice thickness**	3.29 ± 0.80	3.28 ± 0.10	3.32 ± 0.13	t (81.68) = 0.383; *p* = 0.375
**Aneurysm location: n (%)**				
Anterior cerebral artery	13 (11.4)	7 (6.1)	6 (5.3)	X ^2^ (9) = 13.036; *p* = 0.152
Internal carotid artery	21 (18.4)	11 (9.6)	10 (8.8)	
Middle cerebral artery	18 (15.8)	9 (7.9)	9 (7.9)	
Anterior communicating artery	25 (21.9)	11 (9.6)	13 (11.4)	
Posterior communicating artery	9 (7.9)	6 (5.3)	3 (2.6)	
Vertebral artery	6 (5.3)	6 (5.3)	0 (0)	
Posterior cerebral artery	1 (0.9)	1 (0.9)	0 (0)	
Basilar artery	12 (10.5)	11 (9.6)	1 (0.9)	
Superior cerebellar artery	4 (3.5)	3 (2.6)	1 (0.9)	
Posterior inferior cerebellar artery	5 (4.4)	3 (2.6)	2 (1.8)	
**Mean aneurysm size (mm)**	5.10 ± 2.47	5.06 ± 0.30	5.10 ± 0.38	t (88.52) = 0.088; *p* = 0.465
**Hunt and Hess Grade: n (%)**				
1	20 (18.0)	13 (11.7)	7 (6.3)	X ^2^ (4) = 7.428; *p* = 0.115
2	30 (27.0)	18 (16.2)	12 (10.8)	
3	32 (28.8)	19 (17.1)	13 (11.7)	
4	22 (19.8)	9 (8.1)	13 (11.7)	
5	7 (6.3)	7 (6.3)	0 (0)	
**Modified Fisher Grade: n (%)**				
1	1 (0.9)	1 (0.9)	0 (0)	X ^2^ (4) = 4.415; *p* = 0.353
2	8 (7.0)	7 (6.1)	1 (0.9)	
3	15 (13.2)	7 (6.1)	8 (7.0)	
4	86 (77.2)	50 (45.6)	36 (31.6)	
5	2 (1.8)	1 (0.9)	1 (0.9)	
**Aneurysm treatment: n (%)**				
No treatment	7 (6.5)	7 (6.5)	0 (0)	X ^2^ (2) = 4.859; *p* = 0.094
Clipping	52 (48.6)	30 (28.0)	22 (20.6)	
Coiling	48 (44.9)	29 (27.1)	19 (17.8)	
**Intraventricular Hemorrhage: n (%)**	98 (90.8)	60 (87.0)	38 (82.6)	X ^2^ (1) = 0.003; *p* = 0.940
**Intracerebral Hemorrhage: n (%)**	61 (56.5)	31 (44.9)	30 (65.2)	X ^2^ (1) = 5.865; *p* = ** 0.012 ^a^**
**Mean analytical values at the Emergency Department**				
Hemoglobin (g/dL)	13.4 ± 3.1	13.6 ± 0.5	13.3 ± 0.3	t (97.82) = 0.364; *p* = 0.749
Leucocytes (x103 /µL)	13 ± 4	13 ± 1	13 ± 1	t (106.61) = 0.132; *p* = 0.899
Platelets (x103/µL)	233 ± 65	237 ± 10	225 ± 9	U = 1460; *p* = 0.614
C-reactive protein (mg/dL)	12 ± 20	12 ± 3	11 ± 2	t (95.62) = -0.350; *p* = 0.726
Sodium (mEq/L)	139 ± 4	139 ± 1	140 ± 1	t (103.33) = -1.851; *p* = 0.067
Potassium (mEq/L)	3.9 ± 0.5	3.9 ± 0.1	3.8 ± 0.1	t (100.37) = 1.084; *p* = 0.294
Chloride (mEq/L)	105 ± 5	104 ± 1	106 ± 1	t (98.69) = -2.943; *p* = ** 0.004 ^a^**

Abbreviations: X ^2^ , Chi-squared; HU, Hounsfield units.

Notes: The continuous variables, expressed as mean ± standard deviation values, were compared using unpaired *t* -tests or Mann-Whitney *U* tests, as appropriate. The categorical variables, expressed as number (%), were analyzed using the Chi-squared test. ^a^ The values in bold stand for statistically significant differences ( *p* < 0.05).

Significant associations were observed regarding vasospasm and the presence of ICH and the interpeduncular HU value ( *p* = 0.012 and 0.002 respectively). No significant associations were found between vasospasm occurrence and age, sex, aneurysm location, score on the modified Fisher scale, type of treatment, or presence of intraventricular hemorrhage. Although there was a trend toward higher Hunt and Hess grades being associated with vasospasm, it did not reach statistical significance ( *p* = 0.115).

In the subgroup analysis of patients with symptomatic vasospasm, no significant differences were observed in the distribution of HU values ( *p* = 0.820) or chloride levels ( *p* = 0.909) between the symptomatic and asymptomatic groups, nor for the presence of ICH ( *p* = 0.644). These results are detailed in the Supplementary Material (available at https://www.arquivosdeneuropsiquiatria.org/wp-content/uploads/2026/06/ANP-2025.0417-Supplementary-Material.docx ).

The measurement of HU values demonstrated good interobserver reliability (ICC = 0.911; *p* < 0.001). No significant differences were found in CT slice thicknesses between patients with and without vasospasm.

The laboratory analysis, also presented in Table 1 , revealed that serum chloride levels were significantly higher (mean: 106 ± 1 mEq/L; *p* = 0.004) in patients who developed vasospasm. Other parameters, including hemoglobin, leucocyte count, platelet count, C-reactive protein, sodium, and potassium levels, did not show significant differences between groups; however, sodium levels trended toward significance ( *p* = 0.067).

The multivariate logistic regression analysis confirmed an independent association of serum chloride levels on admission with vasospasm (odds ratio [OR] = 3.545; 95%CI = 1.457–8.621; *p* = 0.005). Additionally, the presence of ICH (OR = 2.482; 95%CI = 1.019–6.046; *p* = 0.045) and successive increases in HU in the interpeduncular cistern (OR = 1.253; 95%CI = 1.044–1.504; *p* = 0.016) were independently associated with an increased likelihood of developing this condition.

Given the strength of these associations, we developed a simplified predictive score incorporating these three variables. One point was assigned for interpeduncular HU > 44 (cutoff determined using the ROC curve analysis), and 2 points each for serum chloride ≥ 106 mEq/L and the presence of ICH, based on their higher logistic regression coefficients. The final score ranged from 0 to 5 points. Because only 1 (0,9%) patient had a score of 0, this category was merged with patients scoring 1 for the purposes of statistical analysis and model stability ( Figure 3 ).

**Figure 3:**
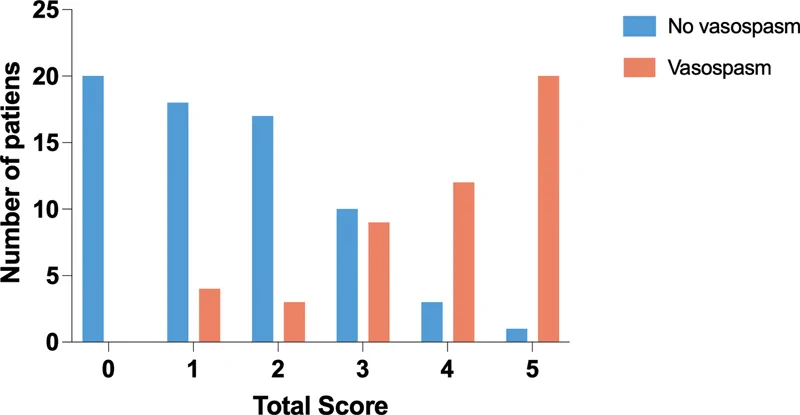
Distribution of the 5-point vasospasm prediction score in patients with and without vasospasm. The graph shows the number of patients at each score level (0–5 points), stratified by vasospasm outcome. Lower scores were predominantly observed in patients without vasospasm, while higher scores (4–5) were more frequent among those who developed vasospasm.

The predicted probability of vasospasm increased with each point in the score: 9.5% with a score of 1, 14.9% with a score of 2, 47.4% with a score of 3, 84.6% with a score of 4, and 95.2% with a score of 5.

This score demonstrated excellent discriminative performance, with an area under the curve (AUC) of 0.886 (95%CI), as illustrated in Figure 4 . Internal validation using 1 thousand bootstrap iterations confirmed the robustness of this model, with a bootstrapped AUC of 0.885 (95%CI: 0.832–0.930). Logistic regression confirmed a significant association between higher score values and vasospasm risk ( *p* < 0.001). Calibration was assessed using the Hosmer-Lemeshow test, which showed a perfect fit (Chi-squared [χ ^2^ ] = 0.000; *p* = 1.000), and the global classification revealed an overall accuracy of 83.5%, with high specificity (95.6%) and a sensitivity of 66.0%.

**Figure 4:**
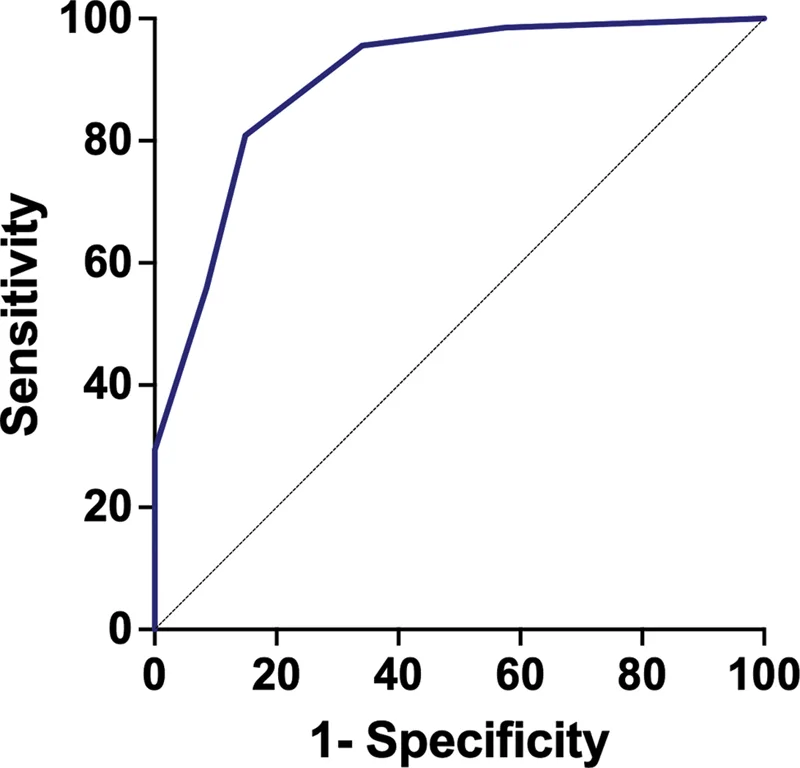
Receiver operating characteristic (ROC) curve for the vasospasm prediction score. The score showed excellent discriminatory performance, with an area under the curve (AUC) of 0.886.

## DISCUSSION

The current study aimed to investigate the relationship between various clinical and radiological parameters and the development of vasospasm in patients with aSAH. We found that ICH, higher interpeduncular HU values, and elevated chloride levels were significantly associated with vasospasm. Moreover, the combination of these parameters demonstrated superior predictive ability for vasospasm compared with each parameter alone, suggesting a synergistic effect in the pathogenesis of this condition.

To enhance clinical applicability, we translated the multivariate model into a simplified 5-point scoring system by grouping and weighting the predictive variables according to their ORs. This scoring approach demonstrated excellent discriminative capacity, with an AUC of 0.886, which enabled the effective risk stratification of vasospasm. Patients with higher scores showed a stepwise increase in the predicted probability, ranging from approximately 9% for a score of 1 to more than 95% for a score of 5. Importantly, this categorical score is more intuitive and easier to implement in clinical practice than raw regression coefficients or complex prediction formulas, and it may enable frontline clinicians to estimate individual risk upon admission and prioritize monitoring, imaging, or preventive strategies accordingly.

Given these findings, establishing a follow-up protocol for high-risk patients could be valuable. Conventional follow-up of aSAH patients, particularly in low-income settings, typically relies on clinical assessments and transcranial Doppler studies to detect vasospasm. However, by the time clinical symptoms and Doppler changes become noticeable, vasospasm is often already well-established, diminishing the effectiveness of therapeutic interventions. 3 7

As no statistically significant differences in HU values or chloride levels were observed between symptomatic and asymptomatic vasospasm in the subgroup analysis and considering the limited sample size of the symptomatic group, these data were combined into a single analysis of angiographic vasospasm. Consequently, we developed a predictive formula, based on ICH, HU > 44, and chloride levels ≥ 106 mEq/L, which ultimately culminated in the creation of a straightforward clinical score that has the potential to identify high-risk patients who could benefit from tailored follow-up strategies. Recent studies, 15 for example, suggest that performing perfusion CT scans on days 3 and 5 postictus could improve prediction accuracy and enable timely therapeutic interventions.

Previous research also supports these findings individually. First, our analysis revealed a significant association between ICH and vasospasm. Regarding pathophysiology, ICH can exacerbate inflammatory responses and secondary brain injury, contributing to vasospasm. Hemorrhagic products likely stimulate an early-phase inflammatory response, enhancing leukocyte and platelet activity, and further promoting vasospasm. 6 16

Second, we confirmed the interpeduncular HU value as a potential predictor of vasospasm, by showing that patients with higher values in the interpeduncular cistern had an increased OR, of 1.253 ( *p* = 0.016), for this condition. This parameter likely reflects the severity and concentration of hemorrhage, as higher HU values indicate denser blood clots, which can correlate with the extent of inflammation and the risk of vasospasm. This finding aligns with those in the existing literature; Ishihara et al., 10 for instance, demonstrated that measuring the HU value in the same location could predict symptomatic vasospasm in patients with aSAH, showing good discriminative power (AUC = 0.83). 10 11 17

Third, we found evidence that chloride levels higher or equal to an established cut-off point (106 mEq/L) can predict the development of vasospasm in patients with aSAH, with a sensitivity of 58% and a specificity of 70%. Elevated chloride levels activate calcium-activated chloride channels in vascular smooth-muscle cells, leading to an influx of calcium that triggers contractions. 18 Additionally, high chloride levels can increase intracellular calcium levels, reinforcing vasoconstriction by promoting thromboxane release and enhancing vascular response to other constrictors. Hyperchloremia can also induce acidosis, associated with neurotoxic effects on neuronal cells, potentially leading to hyperexcitation and neurological damage. Furthermore, inflammatory markers, such as interleukins and the tumor necrosis factor, known to be elevated in acidotic states, have been linked to poorer outcomes and increased risk of vasospasm in aSAH patients. These results suggest that monitoring serum chloride levels can be crucial for the early detection of vasospasm, especially considering the high early mortality rate associated with aSAH. Since chloride levels are modifiable through intravenous fluid composition, early monitoring and tailored fluid therapy may represent an opportunity to influence outcomes. Further research is warranted to determine whether targeted correction of chloride levels could effectively reduce the risk of vasospasm. 19

The association between sodium levels and vasospasm following aSAH remains inconclusive. While several studies have suggested a correlation between hyponatremia and vasospasm, 20 other research, such as that by Chua et al., 21 found no significant correlation. Similarly, in the current study, we did not find a statistically significant association, possibly due to the limited sample size. Nonetheless, managing sodium levels in aSAH patients is considered crucial to prevent complications such as cerebral edema and secondary brain injury. 3 While maintaining appropriate sodium levels is vital for overall patient care, the direct impact of its fluctuations on vasospasm may be less significant compared with factors such as hematoma density and chloride levels.

Despite the fact that multiple inflammatory pathways are activated in the early stages following aSAH, the relationship involving inflammatory markers and vasospasm is not well-established. Elevated white-blood-cell count and HU values have been associated 18 22 with an increased risk of developing delayed cerebral ischemia, the leading cause of mortality and morbidity in patients surviving the ictal phase of aSAH. In delayed cerebral ischemia pathogenesis, inflammation can lead to endothelial dysfunction, increased vascular permeability, and subsequent vasospasm, contributing to ischemic events. 7 However, the findings of the present study did not show a statistically significant association between white-blood-cell count and C-reactive protein levels, which are peripheral markers of inflammation and vasospasm incidence. This aligns with similar findings from other studies 11 that have reported no significant correlation. These divergent findings imply that, while inflammation plays a role in the pathogenesis, its direct relationship with vasospasm may be more complex and influenced by other factors not captured by white-blood-cell count and C-reactive protein levels alone.

Other predictors of angiographic vasospasm have been consistently described in the literature, particularly those reflecting hemorrhage burden and clinical severity on admission. Among imaging-based markers, the extent of subarachnoid blood, which is commonly assessed using the modified Fisher scale, has been repeatedly associated with vasospasm risk, supporting the concept that a higher clot burden increases exposure to blood breakdown products and promotes endothelial dysfunction and prolonged arterial constriction. Likewise, worse clinical grades on admission, such as higher Hunt and Hess scores, have also been linked 11 23 to increased vasospasm incidence, likely reflecting more severe initial injury and greater physiological stress. In the cohort of the present study, although the scores on the modified Fisher scale and clinical grade showed trends toward an association with vasospasm, these relationships did not reach statistical significance, which may be explained by limited statistical power and the multifactorial nature of vasospasm. These findings highlight that, while traditional predictors remain clinically relevant, integrating complementary imaging and biochemical markers, such as interpeduncular HU values and serum chloride levels, may enhance early risk stratification beyond clot burden alone.

The current study has several limitations. First, the relatively small sample size ( *n* = 115) may have limited the ability to detect more subtle associations, and the single-center design may reduce generalizability to other populations and clinical settings, where patient demographics and management practices may differ. In this context, although the scores on the modified Fisher scale appeared to be distributed differently between patients with and without angiographic vasospasm, the study may have been underpowered to detect statistically significant differences for certain established predictors. Therefore, a type-II error cannot be excluded, and clinically-relevant associations reported in the literature may not have reached statistical significance in our cohort.

Second, the retrospective nature of the study introduces potential biases and relies on the accuracy and completeness of medical records. In addition, imaging-acquisition heterogeneity represents a relevant limitation. Although CT-slice thickness was recorded and did not differ significantly between patients with and without angiographic vasospasm, examinations were performed using different scanners and acquisition parameters, which may have introduced residual variability in HU measurements. Nevertheless, HU measurements were independently performed by two experienced raters and demonstrated high interobserver reliability, supporting the robustness of this imaging parameter despite acquisition variability.

Third, treatment-related factors may represent potential confounders. However, all patients were managed according to a standardized institutional protocol, including routine prophylactic nimodipine and standardized management of acute hypertension, which likely reduced treatment-related variability across the cohort. Rescue therapies, such as intravenous milrinone or endovascular verapamil, were administered only after the diagnosis of vasospasm; therefore, it is unlikely that they have influenced its occurrence. Still, a residual influence of treatment-related factors cannot be entirely excluded and is acknowledged as a limitation.

Finally, additional clinical factors, such as initial hemorrhage severity and patient comorbidities, might have influenced vasospasm development and were not fully accounted for. These limitations underscore the need for larger, prospective multicenter studies to validate our predictive model, refine risk stratification strategies in aSAH, and further explore the role of inflammatory markers and other biochemical parameters in vasospasm pathophysiology and prevention.

In conclusion, we have identified significant associations regarding intracerebral hemorrhage, interpeduncular cistern HU values, and elevated serum chloride levels with the development of vasospasm in patients with aSAH. By integrating these variables into a combined predictive model, we have achieved superior accuracy in stratifying patients according to their risk of vasospasm. Based on this model, we have developed, and internally validated, a practical 5-point predictive score designed to support early risk assessment and inform clinical decision-making. This stratification may enable the implementation of tailored monitoring strategies, facilitating earlier detection and timely intervention, with the potential to improve patient outcomes. Multicenter studies are needed to potentially validate these findings and confirm their usefulness in broader clinical settings.

## References

[1] Abraham M K Chang W W Subarachnoid Hemorrhage Emerg Med Clin North Am 2016 34 04 901 916 10.1016/j.emc.2016.06.011 27741994

[2] Long B Koyfman A Runyon M S Subarachnoid hemorrhage: updates in diagnosis and management Emerg Med Clin North Am 2017 35 04 803 824 10.1016/j.emc.2017.07.001 28987430

[3] Etminan N Macdonald R L. Neurovascular disease, diagnosis, and therapy: subarachnoid hemorrhage and cerebral vasospasm. Handb Clin Neurol2021;176:135–169 10.1016/B978-0-444-64034-5.00009-2 33272393

[4] De Rooij N K Linn F HH Van der Plas J A Algra A Rinkel G JE Incidence of subarachnoid haemorrhage: a systematic review with emphasis on region, age, gender and time trends J Neurol Neurosurg Psychiatry 2007 78 12 1365 1372 10.1136/jnnp.2007.117655 PMC2095631 17470467

[5] Etminan N Chang H S Hackenberg K Worldwide incidence of aneurysmal subarachnoid hemorrhage according to region, time period, blood pressure, and smoking prevalence in the population: a systematic review and meta-analysis JAMA Neurol 2019 76 05 588 597 10.1001/jamaneurol.2019.0006 PMC6515606 30659573

[6] Macdonald R L Weir B KA A review of hemoglobin and the pathogenesis of cerebral vasospasm Stroke 1991 22 08 971 982 10.1161/01.STR.22.8.971 1866764

[7] Macdonald R L Delayed neurological deterioration after subarachnoid haemorrhage Nat Rev Neurol 2014 10 01 44 58 10.1038/nrneurol.2013.246 24323051

[8] Østergaard L Aamand R Karabegovic S The role of the microcirculation in delayed cerebral ischemia and chronic degenerative changes after subarachnoid hemorrhage J Cereb Blood Flow Metab 2013 33 12 1825 1837 10.1038/jcbfm.2013.173 PMC3851911 24064495

[9] Park J S Kang H G Hounsfield unit as a predictor of symptomatic vasospasm and hydrocephalus in good-grade subarachnoid hemorrhage treated with endovascular coiling Quant Imaging Med Surg 2023 13 10 6627 6635 10.21037/qims-23-355 PMC10585502 37869270

[10] Ishihara H Oka F Kawano R Hounsfield unit value of interpeduncular cistern hematomas can predict symptomatic vasospasm Stroke 2020 51 01 143 148 10.1161/STROKEAHA.119.026962 31694506

[11] Harrod C G Bendok B R Batjer H H Prediction of cerebral vasospasm in patients presenting with aneurysmal subarachnoid hemorrhage: a review Neurosurgery 2005 56 04 633 654, discussion 633–654 10.1227/01.NEU.0000156644.45384.92 15792502

[12] Steiner T Juvela S Unterberg A Jung C Forsting M Rinkel G European Stroke Organization European Stroke Organization guidelines for the management of intracranial aneurysms and subarachnoid haemorrhage Cerebrovasc Dis 2013 35 02 93 112 10.1159/000346087 23406828

[13] Vergouwen M DI Vermeulen M van Gijn J Definition of delayed cerebral ischemia after aneurysmal subarachnoid hemorrhage as an outcome event in clinical trials and observational studies: proposal of a multidisciplinary research group Stroke 2010 41 10 2391 2395 10.1161/STROKEAHA.110.589275 20798370

[14] Merkel H Lindner D Gaber K Standardized classification of cerebral vasospasm after subarachnoid hemorrhage by digital subtraction angiography J Clin Med 2022 11 07 2011 10.3390/jcm11072011 PMC9000178 35407619

[15] Malinova V Döring K Psychogios M N Rohde V Mielke D Impact of implementing an elaborated CT perfusion protocol for aneurysmal SAH on functional outcome: CTP protocol for SAH AJNR Am J Neuroradiol 2021 42 11 1956 1961 10.3174/ajnr.A7279 PMC8583263 34556476

[16] Sato T Sasaki T Sakuma J Quantification of subarachnoid hemorrhage by three-dimensional computed tomography: correlation between hematoma volume and symptomatic vasospasm Neurol Med Chir (Tokyo) 2011 51 03 187 194 10.2176/nmc.51.187 21441734

[17] Hu P Yang X Li Y Predictive effects of admission white blood cell counts and hounsfield unit values on delayed cerebral ischemia after aneurysmal subarachnoid hemorrhage Clin Neurol Neurosurg 2022 212 107087 10.1016/j.clineuro.2021.107087 34929583

[18] Wray S Prendergast C Arrowsmith S Calcium-activated chloride channels in myometrial and vascular smooth muscle Front Physiol 2021 12 751008 10.3389/fphys.2021.751008 PMC8637852 34867456

[19] Zhou Y Li R Wang K A retrospective study on the relationship between serum electrolyte disorder and delayed cerebral infarction after aneurysmal subarachnoid hemorrhage J Stroke Cerebrovasc Dis 2024 33 04 107579 10.1016/j.jstrokecerebrovasdis.2024.107579 38325032

[20] Uozumi Y Mizobe T Miyamoto H Decreased serum sodium levels predict symptomatic vasospasm in patients with subarachnoid hemorrhage J Clin Neurosci 2017 46 118 123 10.1016/j.jocn.2017.08.037 28887070

[21] Chua M MJ Enríquez-Marulanda A Gomez-Paz S Sodium variability and probability of vasospasm in patients with aneurysmal subarachnoid hemorrhage J Stroke Cerebrovasc Dis 2022 31 01 106186 10.1016/j.jstrokecerebrovasdis.2021.106186 PMC9000130 34749298

[22] Ma X Lan F Zhang Y Associations between C-reactive protein and white blood cell count, occurrence of delayed cerebral ischemia and poor outcome following aneurysmal subarachnoid hemorrhage: a systematic review and meta-analysis Acta Neurol Belg 2021 121 05 1311 1324 10.1007/s13760-020-01496-y PMC7796813 33423218

[23] Frontera J A Claassen J Schmidt J M Prediction of symptomatic vasospasm after subarachnoid hemorrhage: the modified fisher scale Neurosurgery 2006 59 01 21 27, discussion 21–27 10.1227/01.neu.0000243277.86222.6c 16823296

